# Knowledge and perception on type2 diabetes and hypertension among HIV clients utilizing care and treatment services: a cross sectional study from Mbeya and Dar es Salaam regions in Tanzania

**DOI:** 10.1186/s12889-018-5639-7

**Published:** 2018-07-28

**Authors:** Gibson B. Kagaruki, Mary T. Mayige, Esther S. Ngadaya, Andrew M. Kilale, Amos Kahwa, Amani F. Shao, Godfather D. Kimaro, Chacha M. Manga, Doris Mbata, Godlisten S. Materu, Ray M. Masumo, Sayoki G. Mfinanga

**Affiliations:** 1 National Institute for Medical Research, Tukuyu Centre, P. O. Box 538, Tukuyu, Mbeya, Tanzania; 2 National Institute for Medical Research, Headquarters, P. O. Box, 9653 Dar es Salaam, Tanzania; 3 National Institute for Medical Research, Muhimbili Centre, P. O. Box, 3436 Dar es Salaam, Tanzania

**Keywords:** Type2 diabetes/hypertension, Knowledge, Perception, ART and ART Naïve clients, Tanzania

## Abstract

**Background:**

Type2 Diabetes and Hypertension (T2DM/HTN) have become serious threats to the health and socio-economic development in the developing countries. People living with HIV (PLHIV) infection are more vulnerable of developing T2DM/HTN due to HIV infection itself and antiretroviral treatments. The situation is worse when behavioral and biological risk factors are pervasive to PLHIV. Despite this vicious circle; information on the level of knowledge and perception regarding prevention of T2DM/HTN, risks factors and associated complications among PLHIV is not well documented in Tanzania. The aim of this paper was assess the level of T2DM/HTN knowledge and perception among PLHIV and utilizing care and treatment clinic (CTC) services.

**Methods:**

A cross-sectional study was conducted in randomly selected 12 CTCs between October 2011 and February 2012. Data on demographic characteristics, type 2 diabetes and hypertension knowledge and perception were collected from the study participants.

**Results:**

Out of 754 PLHIV and receiving HIV services at the selected CTCs, 671 (89%) consented for the study. Overall 276/671(41.1%) respondents had low knowledge on type2 diabetes and hypertension risk factors and their associated complications. Locality (rural) (AOR = 2.2; 95%CI 1.4–3.4) and never/not recalling if ever measured blood glucose in life (AOR = 2.3; 95%CI 1.1–5.7) were significant determinants of low knowledge among clients on ART. Being currently not having HIV and T2DM/HTN co-morbidities (AOR = 2.2; 95%CI 1.2–4.9) was the only determinant of low knowledge among ART Naïve clients. With regard to perception, 293/671(43.7%) respondents had negative perception on diabetes and hypertension prevention. Sex (female) (AOR = 2.0, 95%CI 1.2–2.9), being aged < 40 years (AOR = 1.6; 95%CI 1.1–2.5) and education (primary/no formal education) (AOR = 4.4; 95%CI 2.0–9.8) were determinants for negative perception among clients on ART while for ART Naïve clients; HIV and T2DM/HTN co-morbidities (AOR = 2.0; 95%CI 1.2–4.6) was the main determinant for negative perception.

**Conclusion:**

Considerable number of respondents had low level of knowledge (41.1%) regarding T2DM/HTN specifically on the risk factors, prevention strategies and their associated complications and negative perception (43.7%) towards healthy practices for mitigating risk behaviors of the diseases. There is need for promoting awareness of T2DM/HTN risk factors and complications by considering determinants of low knowledge and negative perception among PLHIV.

## Background

Non-communicable diseases (NCDs) including type2 diabetes and hypertension have posed a double burden to people living with HIV (PLHIV) in low- and middle-income countries. HIV is a serious public health problem killing potential population group worldwide. Evidence shows that in 2013 about 35.3 million people were living with HIV in the world and about 71 and 70% of HIV infected people and new infections respectively were from Sub-Saharan Africa [1].

Apart from socio-economic impacts; HIV has also been associated with the risk of developing type2 diabetes and hypertension (T2DM/HTN) [2–6]. The linkage between T2DM/HTN and HIV are because HIV itself has been associated with the damage of blood vessels cells and causing atherosclerosis [7, 8]. On the other hand, PLHIV who are on ART have been reported to be at high risk of developing type2 diabetes and hypertension, probably due to: cumulative exposure to ART [9–11], increased risks of cholesterol and fatty acid imbalance which are associated with dyslipidemia and metabolic syndrome [3, 12–15]. Moreover, because of availability of ART, PLHIV in this era are living longer than before and this exposes them to chronic medical conditions such as renal failure (18; 19), degenerative diseases such as T2DM/HTN and their risk factors due the aging processes [16]. Despite improved life expectancy; ART including nucleotide reverse transcriptase Inhibitors (NRTIs) and stavudine based regimens have been reported to increase the risk of raised blood levels of lipid profile (triglycerides, low density lipoprotein cholesterol and total cholesterol) as well as lowering high density lipoprotein [17, 18] which are the risk factors for developing T2DM/HTN. Literature shows that prevalence of hypertension among PLHIV was more than twice that of the general population [19] and that duration on ART is associated with increased cardiovascular risk factors among PLHIV who are on ART [20]. Further, HIV/AIDS, ART and aging among PLHIV have been consistently reported to be associated with renal related morbidity and mortality [21, 22] and type2 diabetes [9–11].

Management of PLHIV with type2 diabetes and hypertension co-morbidities is challenging and costly especially in the settings where resources are limited like Tanzania. Knowledge to PLHIV about their increased risks to develop T2DM/HTN is therefore important in order to help them to engage into the preventive strategies and healthy practice which in-turn may help to prevent or delay the occurrence of these co-morbidities. Additionally, knowledge about the diseases is so crucial because it has been shown to affect practices, disease prognosis and progression, control and management strategies as well as risks avoidance [23, 24]. In addition, gaining or maintaining heavy weight has been perceived by PLHIV as stigma avoidance strategy, an attitude which increase the chance of developing T2DM/HTN [25]. Surprisingly, in Tanzania and in most of low-income countries, there is limited published data on the knowledge and perception on hypertension and type2 diabetes (T2DM/HTN) among PLHIV. This study was therefore designed and conducted to contribute in filling this information gap and also to inform policy and HIV related programs.

## Methods

### Study area and design

This was cross-sectional study conducted among PLHIV aged 18 years and above registered at care and treatment clinics (CTCs) in two purposefully selected regions namely Mbeya and Dar es Salaam in Tanzania. The former representing a rural setting and the latter is the largest urban city in Tanzania. The two regions had the largest number of PLHIV enrolled in CTCs in the country [26]. A total of 12 CTCs were involved in the study. The recruited study participants were interviewed using structured questionnaires and focus group discussions (FGDs) were conducted using an interview guide.

### Data collection

The sample size for this analysis is 745 PLHIV. Estimated using a random sample size calculation formula with the following statistical parameters; “p” the prevalence of diabetes mellitus type2 among HIV positive clients admitted with stroke at Muhimbili National Hospital, 11.1% [27]; Confidence Level: 95%, Z_1-α/2_ = 1.96, marginal of errors (e) = 3%, non response rate of 15% and design effect of 1.5. The study participants were drawn from 12 health facilities – six from Dar es Salaam and six from Mbeya. The details of how these facilities were selected are presented elsewhere [3]. We also conducted eight focus group discussions (FGDs) – two per district. The participants in one FGD included HIV positive clients on ART and the other FGD was with ART Naïve participants.

### Data analysis

Quantitative data analysis was done using Stata version 14 (STATA Corp Inc., TX, USA); whereas; information from FGDs (qualitative data) was coded by themes and analyzed using QSR NVivo version 8. Assessment of level of perception of the respondents was done using eight components and a Likert scale measure with four scores that are; strongly agree, agree, disagree and strongly disagree towards various practices that mitigate the risk of T2DM/HTN and related risk factors. Strongly agree and agree were given a score of zero indicating negative perception score. Disagree and strongly disagree were given a score of “1” indicative of positive perception score. True, False and don’t know responses were used to assess the knowledge level of the respondents against each of 9 questions/statements used. A score of “1” was given for correct answer and “0” for incorrect or don’t know response. The overall knowledge and perception score were obtained by summing the responses, which were expected to range between 0 and 9 and 0 to 8 respectively. Overall level of knowledge and perceptions of clients was measured by using first moment rules (central limit theorem approach) as elaborated by Bati et al. and Tolossa et al. [28, 29].

Low/high knowledge level on risk factors and complications of type2 diabetes and hypertension (T2DM/HTN) plus negative/positive perception level on healthy lifestyle practices for T2DM/HTN prevention was considered if the grand scores of each individual were below or above the mean scores respectively. For knowledge assessment, the mean was “3.94” while for the perception it was “3.1”. Pearson Chi square statistics test was used to compare group differences for categorical variables. Logistic regression was used for modeling multiple factors affecting low level of knowledge and negative perception of the respondents. Unadjusted and Adjusted Odds Ratios (OR) with 95% confidence intervals (CI) are reported. Variables that were considered significant in unadjusted analysis at *p* ≤ 0.2 were also considered for adjusted logistics analysis. Associations and difference between dependent and independent variables were considered statistically significant if *p* < 0.05.

## Results

### Demographic characteristics

A total of 671 PLHIV were enrolled in the study and interviewed. Of these, 354/671 (53%) were on ART. Females constituted about 70% of all study participants. Almost half were from urban settings and three quarters were below 44 years. Majority 87.9% had either primary or no formal education and about 35% were farmers (Table 1).

**Table 1:** Distribution of demographic characteristic of the respondents

Characteristics	Frequency	Percentage
Response rate	671	89.0
Type of respondents		
Clients on ART	354	52.8
ART Naïve clients	317	47.2
Sex		
Male	198	29.5
Female	473	70.5
Locality		
Urban	336	50.1
Rural	335	49.9
Age Group		
18–34	251	37.4
35–44	251	37.4
45–54	117	17.4
55+	52	7.8
Education Level		
No formal education	87	13.0
Primary education	503	75.0
Secondary and above	81	12.0
Marital status		
Couple	319	47.5
Non-Couple	352	52.5
Occupation		
Farmer	234	34.9
Self-employed	224	33.4
Employed	76	11.3
Others	91	13.5
Missing	46	6.9

### Knowledge

Level of knowledge did not differ significantly between the two comparison groups (i.e. those on ART and ART Naïve). Overall 276/671(41.1%) participants had low knowledge on type2 diabetes/hypertension (T2DM/HTN) risk factors, prevention strategies and their associated complications. Majority had low knowledge on the daily recommended portions of fruits and vegetables and their relationship with risk of developing T2DM/HTN 613/671(91.3%). Majority of the participants 581/671(86.6%) were aware of the history of family member with diabetes type2, hypertension and the risk of a person to develop these diseases. ART Naïve respondents had low knowledge on effects of consumption of high fat content diet on health and the risk of developing T2DM/HTN as compared with respondents on ART (26.2% vs 20.3%, *p* = 0.044). About 41% of participants were not aware that being overweight or obese is a risk factor for T2DM/HTN and almost half (324/671; 48.3%) of the participants were not aware that consuming large amounts of alcohol 324/671(48.3%) or table salt 287/671(42.8%) is the risk factor for developing hypertension (Table 2) In addition, about half of participants 329/671 (49.0%), did not know about the protective effect of lifestyle changes (healthy diet, increase physical activity, cessation of smoking/tobacco use) on the prevention of T2DM /HTN.

**Table 2:** Percentage of respondents with low knowledge on diabetes type2/hypertension risk factors and negative perception towards changing against risk behaviors of the diseases among HIV clients utilizing care and treatment services in Tanzania (*n* = 671)

Statement for testing knowledge level	Total, n (%)	Clients on ART, n (%)	ART Naïve clients, n (%)	*P*-value
Having family member with history of diabetes, hypertension is a risk to a person to get these diseases	581(86.6)	302(85.3)	279(88.0)	0.181
Eating fruits less than 5 times a week can lead a person to get hypertension or diabetes	613(91.3)	322(91.0)	291(91.8)	0.403
Eating food with too much cholesterol/fats is healthy for person’s life	155(23.1)	72(20.3)	83(26.2)	0.044
If a person is overweight or obese is at risk to get disease like hypertension or diabetes	277(41.3)	146(41.2)	131(41.3)	0.523
Impotence, amputation, blindness, stroke and renal failure are the outcomes of diabetes	473(70.3)	246(69.5)	227(71.6)	0.303
Lifestyle changes (healthy diet, increase physical activity, cessation of smoking/tobacco use) can help to manage diabetes and hypertension	329(49.0)	171(48.3)	158(49.8)	0.374
Alcoholism is the risk factors for diabetes and hypertension	324(48.3)	167(47.2)	157(49.5)	0.298
Doing physical activities that results in sweating can prevent or delay onsets of diseases like diabetes and hypertension	357(53.0)	184(52.0)	173(54.6)	0.276
Adding salt to cooked food is not good for health because it increases possibility of getting hypertension	287(42.8)	142(40.1)	145(45.7)	0.298
Overall proportion of respondents with low knowledge	276(41.1)	141(39.8)	135(42.6)	0.259
Statement for assessing perception level				
If a person suddenly becomes thin and slender is a sign of running bankrupt	214(31.9)	114(36.0)	100(28.2)	0.020
To become thin and slender is not good because people will consider one to be HIV positive	274(40.8)	134(42.3)	140(39.5)	0.262
If somebody is fat has to maintain his/her body structure otherwise can be considered to be HIV positive	336(50.1)	165(52.1)	171(48.3)	0.186
HIV positive person has to be fat if became thin and slander he/she will be stigmatized	423(63.0)	201(63.4)	222(62.7)	0.458
HIV person should eat food with high in fats & carbohydrate content always so as to increase & maintain heavy weight	372(55.4)	183(57.7)	189(53.4)	0.147
An HIV positive person has to drink alcohol daily in order to reduce stress related to his/her status	87(13.0)	44(13.9)	43(12.1)	0.290
An HIV positive person is not supposed to do any casual work which can lead to sweating	286(42.6)	144(45.4)	142(40.1)	0.095
Smoking is not a problem to HIV positive person as it causes relaxation to an individual	87(13.0)	45(14.2)	42(11.9)	0.217
Overall proportion of respondents with negative perception	293(43.7)	144(45.4)	149(42.1)	0.214

Table 3 reports on the determinants of participants’ low knowledge on T2DM/HTN and their associated risk factors and complications. Low level of knowledge was more among participants from rural settings, who reported to have never/unable to recall if ever they had ever measured blood pressure or glucose during their lifetime and those who were had no T2DM/HTN co-morbidity compared with the counter-groups. Participants on ART from the rural (AOR = 2.2; 95%CI 1.4–3.4) and those who reported to have never or were unable to recall if they had ever have their blood glucose measure in their lifetime (AOR = 2.3; 95%CI 1.1–5.7) were more likely to have low knowledge about risk factors and complications of T2DM/HTN. On the other hand, having no T2DM/HTN co-morbidities among participants who were ART Naïve (AOR = 2.2; 95%CI 1.0–4.9) was the only determinant of low knowledge about risk factors, prevention strategies and complications of T2DM/HTN.

**Table 3:** Unadjusted and Adjusted Odd Ratio (OR) analysis to explore determinants of low knowledge level regarding type2 diabetes/hypertension risk factors and complications

Factor	Total with low knowledge, *n* = 276(%)	Clients on ART		ART Naïve clients	
		Unadjusted OR, 95%CI	Adjusted OR, 95%CI	Unadjusted OR, 95%CI	Adjusted OR, 95%CI
Sex					
Male	80(40.4)	1		1	
Female	196(41.1)	1.1(0.7–1.7)		1.0(0.6–1.6)	
Locality					
Urban	112(33.3)	1	1	1	1
Rural	164(49.0)*	2.4(1.6–3.7)	2.2(1.4–3.4)	1.5(1.1–2.3)	1.2(0.8–2.0)
Age Group					
≥ 40 years	181(42.4)	1		1	
< 40 years	95(38.9)	0.8(0.5–1.2)		1.0(0.6–1.7)	
Marital status					
Couple	167(41.5)	1		1	
Non-Couple	109(40.5)	0.9(0.6–1.4)		1.0(0.6–1.6)	
Education Level					
Secondary and above	31(38.3)	1		1	
No formal or having primary	245(41.5)	1.2(0.6–2.2)		1.1(0.5–2.3)	
Ever measured BP in life					
Yes	111(35.6)	1	1	1	1
No	165(46.0)^†^	1.5(1.0–2.3)	1.1(0.7–1.8)	1.6(1.0–2.5)	1.3(0.8–2.1)
Ever measured Blood Glucose in life					
Yes	13(20.6)	1	1	1	1
No	263(43.3)*	2.9(1.2–6.8)	2.3(1.1–5.7)	3.0(1.2–7.5)	2.1(0.8–5.6)
Currently has HIV & NCD Co-morbidities					
Yes	34(31.5)	1		1	1
No	242(43.0)^‡^	1.2(0.7–2.1)		2.8(1.3–6.0)	2.2(1.2–4.9)

P-value for Chi-squire test results: *p < 0.0001, ^†^ *p = 0.001 and* ^‡^ *p < 0.05, Odd Ratio (OR)*

The results from qualitative data complemented quantitative findings; knowledge on type2 diabetes /hypertension and their associated risk factors was generally low amongst the study participants; as seen in some of quotes below:

“You *find that even these ARVs which we are given has led to rapid increase of our body weight thus you may find yourself suffering from high blood pressure which you didn’t have before”* (*R6-FGD ART Naïve Group* **–**Kyela CTC).

**“***In fact, according to my understanding, these diseases are those which one can develop without acquiring from somebody like diabetes, TB, cancer but you just get them from God.”* (*R10-FGD ART Group* –Buguruni CTC).

The discussants also demonstrated that low knowledge on type2 diabetes and hypertension was due to lack of health education provided at the CTCs:

“We are only taught about the kind of diet we are supposed to eat to maintain our CD4 count and not about NCDs” (*R3-FGD ART Naive Group* **-**Kyela CTC).

*“I think we have not been provided with such health education because when we come to the clinic we just stay here for a short time picking ARVs and have our CD4 count checked, then we leave”* (*R3-FGD ART Group* -Mnazi mmoja CTC).

### Perception

Findings from the assessment of participants’ level of perception towards changing unhealthy behavior practices show that overall, 293/671(43.7%) of the respondents had negative perception. Many believed that PLHIV have to be fat because if one becomes thin s/he will suffer from the HIV related stigma 423/671(63.0%) and also they should eat food rich in fats & carbohydrate always in order either increase or maintain their weights 372/671(55.4%). Both participants who were on ART and those who were ART Naïve perceived “If a fatty person suddenly becomes thin and slender is a sign of running bankrupt” and this significantly was more among the ARV naïve (36.0% vs 28.2%, *p* = 0.02) group (Table 2). This was also observed among the FGDs discussants as quoted hereunder:

*“Normally I ask the doctors why my CD4 count are not increasing despites the fact that I eat good food and my weight has increased tremendously” (R7-FGD ART Group Mwananyamala CTC).*

Table 4 reports on the participants’ perception about the T2DM/HTN and their associated risk factors. Negative perception on T2DM/HTN and their associated risk factors was more among participants: from rural than urban settings (51.3% vs 36.0%, *p* < 0.0001) and those with primary or no formal education than those with secondary and above education (46.9% vs 19.8%, *p* < 0.0001). Respondents who were not aware that there is an association between HIV/ART and type 2 diabetes and hypertension were more likely to have negative perception as compared to those who were aware (45.2% vs 32.5%, *p* < 0.05). Determinants of the negative perception among participants on ART; included female sex (AOR = 2.0, 95%CI 1.2–2.9); age < 40 years (AOR = 1.6; 95%CI 1.1–2.5) and primary or no formal education (AOR = 4.4; 95%CI 2.0–9.8). Negative perception in the ART naïve participants group was more among those who had no T2DM/HTN co-morbidities (AOR = 2.0; 95%CI 1.2–4.6) than among those who had T2DM/HTN co-morbidities.

**Table 4:** Unadjusted and Adjusted Odds Ratio (OR) to explore determinants of negative perception on behavioural change against T2DM/HTN risk factors

Factor	Total with negative perception, *n* = 293(%)	Clients on ART		ART Naïve clients	
		Unadjusted OR, 95%CI	Adjusted OR, 95%CI	Unadjusted OR, 95%CI	Adjusted OR, 95%CI
Sex					
Male	78(39.4)	1	1	1	
Female	215(45.5)	1.8(1.1–2.9)	2.0(1.2–3.2)	0.8(0.5–1.4)	
Locality					
Urban	121(36.0)	1		1	1
Rural	172(51.3)*	1.3(0.8–2.0)		2.9(1.8–4.6)	2.4(1.5–3.9)
Age Group					
≥ 40 years	177(41.5)	1	1	1	
< 40 years	116(46.5)	1.3(0.9–2.1)	1.6(1.1–2.5)	1.3(0.8–2.1)	
Marital status					
Couple	172(42.8)	1		1	
Non-Couple	121(45.0)	1.2(0.8–1.8)		1.0(0.6–1.6)	
Education Level					
Secondary/above	16(19.8)	1	1	1	1
No formal or having primary	277(46.9)*	4.4(2.0–9.7)	4.4(2.0–9.8)	2.7(1.2–6.3)	1.6(0.7–4.0)
Ever measured BP in life					
Yes	132(42.3)	1		1	
No	161(44.8)	1.1(0.7–1.6)		1.1(0.7–1.8)	
Ever measured Blood Glucose in life					
Yes	27(42.9)	1		1	1
No	266(43.8)	0.7(0.3–1.3)		1.9(0.8–4.2)	1.1(0.5–2.7)
Being aware that HIV/ART can cause T2DM or HTN					
Yes	26(32.5)	1		1	1
No	267(45.2)^‡^	1.5(0.8–2.7)		2.3(0.9–5.6)	1.7(0.7–4.5)
Currently has HIV & T2DM/HTN Co-morbidities					
Yes	42(38.9)	1		1	1
No	251(44.6)	0.8(0.5–1.4)		2.7(1.3–5.7)	2.0(1.2–4.6)

P-value for Chi-squire test results: *p < 0.0001 and ^‡^ *p < 0.05, Odd Ratio (OR)*

## Discussion

The current study shows that there is low level of knowledge on type 2 diabetes and hypertension (T2DM/HTN) risk factors and associated complications between both HIV positive clients on ART and ART Naïve. Study participants had low knowledge on the relationship between dietary practices and the risks of getting hypertension or type2 diabetes. Determinants of low knowledge among those who were not on ART included being not having HIV and T2DM/HTN co-morbidity. Further, participants perceived that PLHIV need to keep on weight to avoid stigma and that becoming thin and slender is the sign of running bankrupt. This perception was generally more among participants from rural settings and among those with primary or no formal education. Determinants of poor perception among participants who were on ART included sex (female), age (< 40 years) and having primary level or no formal education whereas having no T2DM/HTN co-morbidity was a determinant of poor perception among ARV naïve participants.

Findings from studies conducted elsewhere also suggest that there is a lack of public awareness and knowledge of various factors related to T2DM/HTN [30–32]. Knowledge of risk factors for any disease including T2DM/HTN is essential for one to make an informed decision [33] and can lead to desired health behavioural change [34]. Finding on the low level of knowledge on T2DM/HTN and their associated risk factors among PLHIV is similar to the findings that have also been reported from different other studies conducted elsewhere [35, 36]. So was the study that was conducted by Al-Shafaee et al. [37] which also reported that community knowledge on type2 diabetes in Oman was suboptimal and that there was lack of awareness of major risk factors for diabetes. Findings from this study and those from other studies conducted elsewhere bring up important evidence on the need to review our guidelines and implementation of health education at primary health care targeting patients with chronic diseases, including HIV, diabetic mellitus and hypertension. Looking at the current Tanzanian national HIV/AIDS management guidelines, PLHIV stand an advantage of receiving the necessary basics on how to avoid risky lifestyles to reduce possibilities of developing chronic diseases such as type2 diabetes and hypertension, as thus one would expect good knowledge of type2 diabetes and hypertension among CTCs clients especially those on ART [38].

Association between chronic diseases burden, sedentary lifestyles and consumption of diets that have high amounts of saturated fats, salt and sugar has been established [39]. Our findings show that ART naïve participants were less likely to know the risks associated with the consumption of foods with high fats/cholesterol compared to those who were on ART. They were also less likely to know the protective effect of lifestyles changes such as taking healthy diets, increase physical activity, cessation of smoking/tobacco against type2 diabetes and hypertension. Our findings are also similar to those from another study conducted to assess risk behavioural and environmental factors among non-diabetics attending primary health care centres in Karachi, on knowledge regarding cause, sign and symptoms and complications which reported that practices regarding diet and life style were also unsatisfactory [34]. Literature shows that simple lifestyle modifications, such as a healthy diet that includes reducing sugar intake and appropriate consumption of fruits are considered essential for the prevention and control of type2 diabetes and hypertension [40]. This is contrary from what we found in our study in which the study participants had a perception of eating more carbohydrate foods to gain weight to avoid HIV related stigma and not be seen as bankrupt. This therefore calls for an urgent need to improve dietary education at our CTCs and having an integrated care model where PLHIV can access non communicable diseases (NCDs) care and vice versa. There is also need to increase public awareness regarding modifiable risk factors and healthier lifestyles, and developing strategies to identify and manage at-risk populations. Individuals, communities and relevant authorities may have to think on the best was of translating into action the results reported by Abdesslam Boutayeb and Saber Boutayeb [41] that there is sufficient scientific evidence to suggest that by changing to a healthier diet up to 80% of cases of coronary heart disease, 90% of type 2 diabetes cases, and one-third of cancers could be avoided. Thus, increasing public awareness regarding modifiable risk factors for T2DM/HTN and healthier lifestyles, and developing strategies to identify and manage at-risk populations, are among of the various possible mechanisms being recommended and used to stem the epidemics.

Non communicable diseases have been influenced by industrialization, urbanization a access of different varieties of foods from different corners in the world [39] and literature shows that type2 diabetes and hypertension were uncommon in rural communities [42, 43]. Our findings also show that being rural dwellers, those who have not been previously screened for T2DM/HTN were predictors of low knowledge among those who were on ART. This supports well our results, as there is increased likelihood of disease determinants to be prevalent in areas and communities where the disease prevalence was low and practice do not match with their prevention hence the need for prevention interventions targeting these communities. Our findings are also similar to those reported from another study conducted in Senegal also showed that type2 diabetes and hypertension were frequent among HIV patients on ART [44].

Al-Shafaee et al. [37] reported that higher level of education, a higher household income, and the presence of a family history of type2 diabetes were positively associated with more knowledge. Level of education was also the most significant predictor of knowledge regarding risk factors, complications and the prevention of type2 diabetes [37]. Control of non-communicable diseases requires community knowledge and understanding on how these diseases can be developed and strategies to mitigate exposure to the risks [23, 24]. In the current study respondents had negative perception towards changing practices to reduce risk of diabetes type2/hypertension-related behaviors. Major perceptions were centered on beliefs that PLHIV have to be fat and should always eat high fat and carbohydrate diets to increase and maintain weight as the way of avoiding stigma. The mentality of maintaining hefty weight was linked with increasing level of CD4 counts and perceived as being healthy. Similar finding for desire for weight gain has also be reported in Senegal [25] and South Africa [45] among PLHIV's. Negative perception was more pronounced among rural respondents than the urban counterparts, those with primary or no formal education than those with secondary and above education. Our findings point out that the most significant predictor of knowledge and perception on the risk factors for T2DM/HTN, their complications and prevention is the level of knowledge that an individual has.This therefore raises optimism that health education could be a powerful tool towards preventing and controlling these co-morbidities among PLHIV in the country.

## Conclusion

The level of knowledge among PLHIV on type2 diabetes and hypertension and their associated risks and complications was generally low. It was also observed that significant number of respondents had negative perception towards healthy practices for mitigating risky behaviors for type2 diabetes and hypertension. Maintaining and gaining weight was perceived as way of HIV related stigma avoidance and of not been seen as a bankrupt. The poor knowledge and wrong perception could affect outcomes on interventions for control these type2 diabetes and hypertension co-morbidities among PLHIV. There is therefore, need for continued health education at CTCs as well as promoting awareness creation interventions regarding risk factors, prevention strategies and complications of these co-morbidities.

## Abbreviations

AIDS: Acquired Immunodeficiency Syndrome
ART: Antiretroviral Treatment
CD4: Cluster of Differentiation 4
CI: Confidence Interval
CTC: Care and Treatment Centre
DM: Diabetes Mellitus
FGD: Focus Group Discussion
HIV: Human Immunodeficiency Virus
HTN: Hypertension
NCD: Non communicable diseases
OR: Odds Ratio
PLHIV: People Living with HIV
